# Risk of Methicillin-Resistant *Staphylococcus aureus* (MRSA) and Vancomycin-Resistant *Enterococcus* (VRE) Acquisition in Ambulances: A Retrospective Propensity Score-Matched Cohort Analysis

**DOI:** 10.1017/ash.2021.146

**Published:** 2021-07-29

**Authors:** Diego Schaps, Deverick Anderson, Andrew Godfrey

## Abstract

**Background:** Infection following ambulance transport, or medical-transport–associated infection (MTAI), is understudied. Although medical-transport vehicles are routinely contaminated with methicillin-resistant *Staphylococcus aureus* (MRSA) and/or vancomycin-resistant *Enterococcus* (VRE), an association between vehicle exposure and disease development has not been identified. We estimated the relative risk (RR) of developing MRSA or VRE colonization or infection within 30 days of ambulance exposure. **Methods:** We performed a retrospective cohort study of patients with a principal diagnosis of chest pain presenting to our emergency department (ED) from January 1, 2016, through December 31, 2019. To control for confounding by healthcare exposure, patients were included if they presented from and were discharged to nonhealthcare locations without being admitted to the hospital. Encounters were stratified by whether the patient arrived at the ED via ambulance or private vehicle. Propensity scores were calculated using multivariable logistic regression with ambulance exposure as the dependent variable. Age, smoking status, history of myocardial infarction, congestive heart failure, peripheral vascular disease, cerebrovascular disease, dementia, diabetes mellitus, and chronic kidney disease were included as covariates because their standard differences were >0.10. Propensity score matching was performed in a 2:1 ratio, but not all exposed patients received 2 matching unexposed patients due to a low sample size. A multivariable logistic regression was performed on the matched cohort to estimate the RR of newly diagnosed MRSA or VRE infection or colonization within 30 days following ambulance exposure. **Results:** In total, 321,229 patients had ED encounters during the study period. After applying inclusion criteria and propensity score-matching there were 11,324 patients: 3,903 in the ambulance group and 7,421 in the unexposed group. Moreover, 12 patients (0.11%) had the outcome of interest, including 9 (0.08%) with MRSA and 3 (0.03%) with VRE. The 30-day prevalences of MRSA and VRE were larger in the ambulance group than in the unexposed group: 8 (0.20%) and 4 (0.05%), respectively (*P* = .02). Patients who presented to the ED via ambulance were almost 4 times more likely to have MRSA or VRE within 30 days of their encounter (RR, 3.72; 95% CI, 1.09–12.71; *P* = .04). The RRs for MRSA and VRE alone were 3.33 (95% CI, 0.79–13.94; *P* = .10) and 4.14 (95% CI, 0.37–46; *P* = .25), respectively. **Conclusions:** To our knowledge, our cohort study is the first to demonstrate an association between ambulance exposure and the development of disease. These results represent the first step in evaluating MTAI burden to eventually develop targeted interventions with the purpose of reducing it.

**Funding:** No

**Disclosures:** None

